# Variation spectra in mild isolated hyperthyrotropinemia: pilot cohort and systematic review

**DOI:** 10.3389/fendo.2025.1612450

**Published:** 2025-11-04

**Authors:** Valentina Ricci, María E. Masnata, María D. Villanueva Gonzalez, Rosa E. Enacán, Agustín Izquierdo, Ezequiela Adrover, María Esnaola Azcoiti, Gabriela Sansó, Paula A. Scaglia, Carina M. Rivolta, Héctor M. Targovnik, Rodolfo A. Rey, María G. Ropelato, Ana E. Chiesa, Juan P. Nicola, Mariana L. Tellechea

**Affiliations:** ^1^ Centro de Investigaciones Endocrinológicas “Dr. César Bergadá” (CEDIE) CONICET – FEI – División de Endocrinología, Hospital de Niños Ricardo Gutiérrez (HNRG), Buenos Aires, Argentina; ^2^ Unidad de Medicina Traslacional, Hospital de Niños Ricardo Gutiérrez (HNRG), Buenos Aires, Argentina; ^3^ Cátedra de Genética. Departamento de Microbiología, Inmunología, Biotecnología y Genética. Facultad de Farmacia y Bioquímica. Universidad de Buenos Aires. Hospital de Clínicas “José de San Martín”, Buenos Aires, Argentina; ^4^ Instituto de Inmunología, Genética y Metabolismo (INIGEM). CONICET-Universidad de Buenos Aires. Hospital de Clínicas “José de San Martín”, Buenos Aires, Argentina; ^5^ Departamento de Bioquímica Clínica, Facultad de Ciencias Químicas, Universidad Nacional de Córdoba, Córdoba, Argentina; ^6^ Centro de Investigaciones en Bioquímica Clínica e Inmunología, Consejo Nacional de Investigaciones Científicas y Técnicas (CIBICI-CONICET), Córdoba, Argentina

**Keywords:** hyperthyrotropinemia, next generation sequencing, single nucleotide variants, subclinical hypothyroidism, systematic revision, thyroid dyshormonogenesis, congenital hypothyroidism, genetic diagnosis

## Abstract

**Background:**

Lower thyrotropin (TSH) cutoffs for Congenital Hypothyroidism (CH) during the neonatal period and childhood have led to increased detection of Mild Isolated Hyperthyrotropinemia (MIH) or Subclinical Hypothyroidism; however, genetic testing has been limited in this setting. We aimed to evaluate the contribution and molecular spectrum of genetic variants in MIH.

**Methods:**

Ten patients underwent targeted Next-Generation Sequencing (NGS). Data was analyzed for Single Nucleotide Variants (SNVs), short insertions/deletions, noncanonical splice site (NCSS) variants, and Copy Number Variants (CNVs) in 13 candidate genes associated with thyroid dyshormonogenesis and isolated thyroid hypoplasia. To provide an expanded view of the genes and variants associated with MIH, we performed a Systematic Review (SR) and variant reclassification.

**Results:**

Eight monoallelic SNVs affecting 4 genes were identified in 5 subjects. A potential digenic or pseudo-digenic inheritance was identified in 3 infants. One novel variant was found in the *TG* gene. Genetic diagnosis, established based on the inheritance pattern, zygosity, pathogenicity of the variant, and genotype-phenotype correlation, was highly suggested in 4 patients. Through SR, we created a valuable database resource of 122 unique reclassified SNVs comprising 173 patients.

**Conclusion:**

Results provide further evidence for the elucidation of the genetic etiology of MIH and expand the phenotypic and variant spectrum of CH. Future, more extensive prospective studies are needed to investigate the utility of NGS in guiding treatment decisions and predicting prognosis for MIH patients.

## 1 Introduction

Congenital hypothyroidism (CH) is the most common endocrine disease of newborns. Its incidence varies between 1:2000 and 1:4000 in the world (1). Many countries conduct newborn screening (NBS) tests for CH, resulting in a rapid diagnosis and appropriate treatment for numerous patients, thus preventing neurodevelopmental, growth, and behavioral problems associated with untreated CH. The nationwide NBS program of CH, implemented in 1990 in Argentina, identifies children with high TSH levels, with an incidence of 1:2,367 (2).

The incidence of CH has doubled over the last two decades, particularly in cases with gland-*in-situ* (GIS). Several factors have been proposed to explain this upward trend, including the lowering of the thyrotropin (TSH) cut-off value for CH, the increased survival of extremely preterm infants, who are at greater risk for CH, and the higher prevalence of consanguineous families in several countries (3, 4).

The etiology of primary CH has been traditionally classified as thyroid dysgenesis (TD) or thyroid dyshormonogenesis (TDH). TDH occurs due to any defect in the steps of hormone biosynthesis and usually presents with a structurally normal-sized or goitrous thyroid. TDH constitutes 10–15% of CH patients and is often familial. TD usually occurs because of an ectopic thyroid or athyreosis, with thyroid hypoplasia as the least common cause (5, 6).

Subclinical hypothyroidism (SCH) or Mild Isolated Hyperthyrotropinemia (MIH) is usually defined as a serum TSH concentration above the biochemically defined upper limit of the reference range when serum-free T4 (FT4) concentration is within its reference range and characterized by a normal-sized or hypoplastic thyroid gland (7, 8). Of note, in the literature, the definition of SCH, and even that of CH, can be arbitrary and vary between studies.

Newborns with MIH may have transient impairment of thyroid function due to environmental or mother-derived causes. During childhood, the main cause of persistent MIH is autoimmune disease. Iodine deficiency, obesity, non-thyroidal chronic diseases, or inherited syndromes may also be responsible for mild increases in TSH levels. Persistent MIH may be the expression of mild persistent thyroid failure due to genetic abnormalities of thyroid structure or function.

The studies on the genetics of CH have increased with the advent of Next-Generation Sequencing (NGS), which allows simultaneous analysis of known and candidate genes associated with various diseases. Recent NGS studies have been useful in elucidating the genetic causes of cases of CH (9–13) with a diagnostic yield ranging from 20 to 65% (13–15). Current consensus guidelines on CH recommend that genetic testing should aim to improve the patient’s diagnosis, treatment, or prognosis (16).

*DUOX2* (OMIM: 606759), *TG* (OMIM: 188450), and *TPO* (OMIM: 606765) are the genes most frequently implicated in cases of TDH; however, other genes have also been included in the “Congenital Hypothyroidism: A 2020–2021 Consensus Guidelines Update” as genes associated with TDH, such as *DUOX1*, *DUOXA2*, *SLC5A5* (*NIS*), *SLC26A4* (*PDS*), *SLC26A7*, *IYD* (*DEHAL1*), *GNAS*, and *TSHR* (OMIM: 603372) (16). *DUOX2* variants appear to be the most common cause of permanent TDH in East Asia (13, 17–19). Instead, studies on the Caucasian population showed that *TPO* or *TG* variants are the most common cause of TDH (10, 14). Biallelic variants in *TG* and *TPO* may result in goiter and mild to severe CH (11, 20, 21). Homozygous or compound heterozygous variants in the *TPO* gene lead to a variable degree of iodine organification deficiency characterized by hypothyroidism with elevated thyroglobulin levels (22).

Of note, the *TSHR* gene has a special place in the etiology of primary CH and is involved in TD, TDH, and Resistance to TSH. This last condition of reduced sensitivity to TSH is characterized by elevated serum TSH concentration in the absence of goiter, with a normal or hypoplastic thyroid gland, and normal to very low levels of thyroid hormones (23). Patients with *TSHR* variants present a phenotype varying from persistent MIH with normal-sized glands to severe TD with orthotopic hypoplasia (14, 24–26).

Isolated thyroid hypoplasia may occur because of variants in *PAX8* or *TSHR (* 5 *).* Although thyroid morphology and degree of biochemical hypothyroidism are very variable in the context of *PAX8* variants, patients have been described with MIH or isolated hypoplasia (27). Variants in the transcription factors *NKX2–1* and *FOXE1* cause CH in association with more extensive developmental syndromes (5).

With the widespread use of NBS programs and the application of NGS testing, it has become evident that the genetic architecture of CH is more complex than previously anticipated. A few CH cases appear to be monoallelic, associated with milder phenotypes (10, 11). For *DUOX2* and *TSHR* authors have applied both autosomal recessive and autosomal dominant inheritance (10). Data on oligogenic combinations with one or more variants in different genes in CH have started to accumulate in scientific literature (28).

This study aimed to explore the underlying genetic etiology of MIH in a well-characterized pilot prospective cohort, using a 13-gene virtual NGS-based panel for variant detection. Additionally, to increase the knowledge on the MIH variation landscape and provide insights into the pathogenesis of the disease, we performed a systematic review of variants reported as causal for MIH in scientific literature up to 8 January 2025. Variants were systematically collected, and pathogenicity was reassessed.

## 2 Materials and methods

### 2.1 Ethics statement

The studies involving humans were approved by the Ethics Committee of the Hospital de Niños Dr. Ricardo Gutierrez (HNRG), Buenos Aires, Argentina. The study was carried out following The Code of Ethics of the World Medical Association (Declaration of Helsinki), local legislation and institutional requirements. The participants’ legal guardians/next of kin provided written informed consent for participation in this study.

### 2.2 Study design and setting

We performed a prospective study of a cohort of children diagnosed with MIH at Ricardo Gutiérrez Children’s Hospital (HNRG), a tertiary pediatric public hospital in Buenos Aires, Argentina. Patients diagnosed in HNRG, and those who have been referred to the Hospital with a presumptive or confirmed diagnosis for care, and who meet the defined inclusion and exclusion criteria, were invited to participate in the genetic study. Patients were prospectively recruited between 2018 and 2023 and followed at the HNRG. All study subjects were routinely evaluated for serum TSH, T3, T4, and free T4 (FT4) levels using electro-chemiluminescent immunoassays (ECLIA, Roche Diagnostics GmbH, Mannheim, Germany). Thyroid morphology was evaluated by neck ultrasonography and/or scintigraphy.

The initial calculation of the study sample size was performed to determine the proportion of patients with definitive or inconclusive genetic diagnoses in a longitudinal cohort of children diagnosed with CH. All the patients of the longitudinal study who had MIH were included in the present study. Since this is an interim study of the whole cohort, a specific sample size calculation was not performed.

### 2.3 Patients

Inclusion criteria were: 1) diagnosis of MIH characterized by elevated serum TSH levels between 9 and 20 µU/ml during the neonatal period (up to 3 months of age) or between 5 and 20 µU/ml during childhood, with normal FT4 level for age; and 2) parents or legal guardians agreed to participation and signed the informed consent form. Exclusion criteria were: 1) Known underlying causes of transient thyroid dysfunction, such as prematurity, Down syndrome, maternal thyroid disease, mothers and/or patients with positive serum levels of anti-thyroperoxidase and/or anti-thyroglobulin antibody, and excessive maternal iodine intake; 2) cases with goitrous thyroid gland thyroid and those with TD due to ectopy and athyreosis; and 3) cases with congenital syndromes identified at or before birth.

### 2.4 Outcome measure

The main outcome measure of the study was identifying and characterizing genetic variants (disease-causing variant status).

The genomic DNA was extracted from the peripheral venous blood cells as previously described (29). The DNA was quantified using a high-performance microvolume spectrophotometer NanoPhotometer^®^ NP60 (Implen Inc.), and the DNA concentration was normalized to 10 ng/μL using the Qubit^®^ 3.0 fluorometer (Invitrogen). DNA purity was assessed by measuring the absorbance ratio 260/280 nm; further DNA sample processing was performed only if the ratio was between 1.8 and 2.1.

Different methods for DNA library preparation and hybridization were used: TruSight ™ One (Illumina) in 2 cases, Custom SureSelect X (Agilent) in 2 patients, and Twist Custom Panel in 6 patients. The quality of genomic DNA fragmentation was verified using a capillary system Fragment Analyzer™ (Agilent). NGS by synthesis with fluorescent reversible terminator deoxyribonucleotides was performed using a NextSeq 500^®^ system (Illumina) at the Translational Medicine Unit of the HNRG.

For sequencing data processing, we followed the best practice recommendations from Broad Institute using the Genome Analysis Toolkit (GATK). The demultiplexed fastq files were aligned with GRCh38 reference genome using the BWA-MEM algorithm of Burrows–Wheeler Aligner software. Duplicates were removed using Picard (Broad Institute). Variant Call Format (VCFs) files were annotated with Franklin by Genoox (https://franklin.genoox.com).

We used a phenotype-driven virtual panel including 13 candidate genes: *DUOX2*, *TG*, *TPO*, *DUOX1, DUOXA1*, *DUOXA2*, *SLC5A5*, *SLC26A4 (PDS)*, *SLC26A7*, *IYD (DEHAL)*, *GNAS*, *TSHR*, and *PAX8*. The level of gene-disease association, as described in the standard operating procedure document provided on the ClinGen website (30), was previously evaluated by Feng Sun and col. (17), for all included genes except for *SLC26A7*. They reached clinical validity classifications as definitive for *DUOX2*, *TG*, *TPO*, *DUOXA2*, *SLC5A5*, *SLC26A4*, *IYD*, *TSHR*, and *PAX8*; strong for *GNAS*; moderate for *DUOXA1* and *DUOX1*. *DUOXA1* was previously reported as a possible causative gene for TDH (31).

Data was analyzed for single-nucleotide variants (SNVs) and small indels. The target region included the coding exons, consensus splice sites (± 2 bases from the start or end of an exon), and the +-3->10 splice region.

SNV and small indels were filtered through the Franklin Genoox platform for suggested classification. The pathogenicity of variants was manually established using the American College of Medical Genetics and Genomics (ACMG) and the Association for Molecular Pathology (AMP), and the ClinGen Sequence Variant Interpretation Working Group recommendations. No specific recommendations currently exist for variant interpretation of CH-associated genes. We performed gene- and disease-specific modifications to the framework for variants related to MIH. Supporting information on how we applied the criteria to guide future variant classification is available at **Supplementary Material S1** (32–34).

A Bayesian scoring algorithm modified from Tavtigian et al. (34) was used, and Bayesian scores were binned into the following categories: 0= Benign (B); 0.001-0.051= Likely Benign (LB); 0.100-0.188= VUS (variant of uncertain significance) leaning to Benign (VUS_LB); 0.325-0.500= VUS; 0.675-0.812= VUS leaning to Pathogenic (VUS_LP); 0.900-0.988= Likely Pathogenic (LP); 0.994-0.999= Pathogenic (P).

An explorative analysis of noncanonical splice sites (NCSSs) was performed using the deep-learning tool SpliceAI to identify putative variants causing splice defects. Copy Number Variants (CNVs) were predicted using the coverage-based DECoN (Detection of Exon Copy Number) algorithm (35).

Integrative Genomics Viewer (IGV v.1.4.2) (36) was used to visually inspect the variants. The Human Genome Variation Society (HGVS) nomenclature was checked using Mutalyzer 3 (37).

Putative disease-causing variants were confirmed in patients and parents using Sanger sequencing. Target exons were amplified by polymerase chain reaction (PCR) with specific primers and GoTaq^®^ DNA Polymerase (Promega). The products were sequenced using an ABI 3500 Genetic Analyzer (Applied Biosystems) at the Translational Medicine Unit of the HNRG. The sequences were compared to the reference sequence and analyzed using Chromas (Technelysium Pty Ltd.).

All cases were discussed in rounds of meetings with clinicians and trained molecular geneticists.

### 2.5 Systematic review of sequence variants in MIH and reclassification

The search strategy, eligibility criteria, data extraction, and variant reclassification procedure are available in **Supplementary Material S2**.

## 3 Results

### 3.1 Diagnostic performance in our pilot cohort

In the present study, we included the first ten unrelated children, born full-term to non-consanguineous parents, who entered the study. The clinical characteristics are presented in **Table 1**. Five patients were examined in or near the neonatal period after NBS tests. Five cases had persistently elevated TSH (>5 µU/ml) during follow-up (patients 1, 2, 3, 7, and 9), while the other three had blood levels fluctuating between 3.2 and 11.9 µU/ml throughout the observation period (cases 5, 6, and 8). Patients 4 and 10 are still receiving levothyroxine (L-T4) and have not been re-evaluated yet. In patient 7, levothyroxine withdrawal was attempted, but treatment had to be restarted. A brief report on patient 9, our paradigmatic case, is presented at **Supplementary Material S3** to exemplify the clinical course of MIH.

**Table 1:** Clinical characteristics of the patients.

Patient	Sex	NBS	Age at diagnosis	TSH (µU/ml)	T4 (µg/dl)	FT4 (ng/dl)	Thyroid morphology (US/scan)†	L-T4‡			Last visit	
								Begining	End		Age	TSH (µU/ml)
1	M	NAV	3m	13	10,1	1,4	US: normal	No L-T4			6m	12,9
2	M	NAV	5y 10m	12,5	7,4	NAV	NAV	No L-T4			7 y	8,6
3	F	NAV	1y 6m	14,8	9,9	NAV	US: normal	1y 6m		4y 10m	4y 10m	10,7
4	M	NAV	3y 10m	10,4	NAV	1,1	NAV	5y		Not yet	7y 6m	5,0§
5	M	+	18d	13,4	12,6	1,7	scan: eutopic	9m		3a 4m	5y	6,0
6	F	+	10d	17,2	12,8	1,8	scan: eutopic	No L-T4			2y	6,5
7	F	NAV	8y	8,3	12,9	NAV	US: normal	8,3		Not yet	17y	10,3
8	F	+	1m 11d	10,7	10,5	1,4	scan: eutopic	2m		7y 5m	7y 5m	3,2
9	F	+	1m 7d	19,9	8,8	1,3	scan: eutopic, increased in size. Revaloration US: normal	1m 7d		13y	17y	7,8
10	F	+	21d	20,0	9,9	1,1	scan: eutopic	21 d		Not yet	1y 7m	0,4§

NAV, not available; F, female; M, male; m, months; y, years; d, days; NBS, newborn screening; US, ultrasound; scan, scintigraphy. † In 2 cases thyroid morphology was not evaluated by neck ultrasonography or scintigraphy. ‡ Some infants were treated from diagnosis by levothyroxine (5–15 μg/kg/d) and re-evaluated after three to five years. Patient 7 was diagnosed and treated at 8 and reassessed at 17 years. § TSH level under treatment.

Eight monoallelic SNVs affecting *TSHR*, *DUOX2, TG*, and *TPO* genes were identified in 5 subjects (**Table 2**). Potential digenic or pseudo-digenic combinations were identified in 3 infants. One novel variant was found in the *TG* gene. No clinically significant NCSS variants or CNVs were prioritized. No causative variants were identified in the remaining 9 genes.

**Table 2:** Detailed information of the single nucleotide variants (SNVs) identified.

Patient	Gene	SNV†	ACMG/AMP criteria and classification		Conclusion
2	*TSHR*_(M)_	81139800 NM_000369.5:c.814C>G NP_000360.2:p.Leu272Val	PM2_Supp, PP3_Mod, PP4, PP1	VUS_LP	Inconclusive-Possibly solved
	*TPO*_(F)_	1496041 NM_001206744.2:c.2059G>T NP_001193673.1:p.Glu687Ter	PVS1, PM2_Supp,	LP	
5	*TG*_(M)_	**133131829** **NM_003235.5:c.7880A>G** **NP_003226.4:p.Asp2627Gly**	PM2_Supp, PP3_Supp, PM3	VUS_LP	Inconclusive
6	*DUOX2*	45101227 NM_001363711.2:c.2895_2898del NP_001350640.1:p.Phe966Serfs*29	PVS1, PS3_Supp,	LP	Solved
	*TG*_(F)_	132882609 NM_003235.5:c.886C>T NP_003226.4:p.Arg296Ter	PM2_Supp, PVS1, PM3_VS	P	
8	*TPO*	1542420GG NM_001206744.2:c.2752_2753del NP_001193673.1:p.Ser918Cysfs*62	PM2_Supp, PVS1_Mod	VUS	Inconclusive-Possibly solved
	*TSHR*_(M)_	81143388 NM_000369.5:c.1330T>C NP_000360.2:p.Tyr444His	PM2_Supp, PM1_Supp, PP3, PP4	VUS_LP	
9	*TSHR*_(F)_	81143633 NM_000369.5:c.1575C>A NP_000360.2:p.Phe525Leu	PM2_Supp, PM1, PP4, PS3_Supp, PM5	LP	Solved

† Genomic position (Genome Assembly: GRCh38) and description using HGVS guidelines. ACMG-AMP: American College of Medical Genetics and Genomics - Association for Molecular Pathology. *TSHR* (NG_009206.1): thyroid-stimulating hormone receptor. *TPO* (NG_011581.2): thyroid peroxidase. *TG* (NG_015832.2): thyroglobulin. *DUOX2* (NG_009447.1): dual oxidase 2. P, Pathogenic; LP, Likely Pathogenic; VUS_LP, Variant of Uncertain Significance leaning to Pathogenic; VUS, Variant of Uncertain Significance. All variants were confirmed by Sanger sequencing in the patients and their parents, with a few exceptions (**Supplementary Figure S5**). (M) maternally inherited. (F) paternally inherited. In bold a novel variant.

Genetic diagnosis criteria for MIH were established considering the inheritance pattern, the zygosity, the pathogenicity of the variant, the segregation analysis, and the phenotypic specificity according to international recommendations and the results of our systematic literature review (**Supplementary Table S4**). According to ACMG/AMP guidelines, only variants classified as P or LP are considered definitive for a genetic diagnosis. Accordingly, cases 6 and 9, heterozygous carriers of LP variants in *DUOX2* and *TSHR* genes, respectively, were considered genetically solved (**Table 2**). Meanwhile, patients 2 and 8, heterozygous carriers of VUS_LP variants in the *TSHR* gene were considered potentially solved. The genetic etiology remained ambiguous for patient 5 since the contribution of the monoallelic *TG* variant is unclear. The remaining patients, for whom no disease-causing variants were identified, were classified as unsolved.

### 3.2 Systematic review

The search and selection process, including the Flowchart of manuscript selection, is available in **Supplementary Material S6.1–4**. Forty-four articles were included (**Supplementary Material S6.3**). The “Mild Isolated Hyperthyrotropinemia Variants Database” is openly available at http://hdl.handle.net/11336/254971.

**Table 3** summarizes data, creating a representative overview of the published MIH data. We found 215 variants comprising 45 articles and 173 patients, mainly in 4 genes, as expected: *TSHR*, *DUOX2*, *TPO*, and *TG*. Eight additional sequence variants were identified in other genes in the setting of MIH (*DUOX1*, *DUOXA2*, *SLC26A4*, *PAX8*, and *GLIS3*). After deduplication, only 122 unique variants remained. When we assessed the prevalence of reported variants in MIH, the *TSHR* gene was the most frequently mutated gene with 51 variants, accounting for 45% of the total variation in MIH. *DUOX2* followed with 32 variants.

**Table 3:** Summary of unique reclassified variants.

Variant reclassification verdict	*TSHR*	*DUOX2*	*TPO*	*TG*	All
Gene					
All	51 (45)	32 (28)	13 (12)	17 (15)	113†
P	3	14	2	1	20 (18)
LP	29	6	4	1	40 (35)
VUS_LP	13	3	4	3	23 (20)
Other	6	9	3	12	30 (27)

The numbers represent the count of variants. In parenthesis the proportion (%). † Nine monoallelic variants were also reported in the following genes: *GLIS3*, *SLC26A4*, and *PAX8* (not shown). *THSR*, thyroid-stimulating hormone receptor; *TPO*, thyroid peroxidase; *TG*, thyroglobulin; *DUOX2*, dual oxidase 2; P, Pathogenic; LP, Likely Pathogenic; VUS_LP, Variant of Uncertain Significance leaning to Pathogenic.

According to variant type, missense variants represent the major set at 61% (N = 51), followed by frameshift (N = 16) and nonsense (N = 8) variants. Splicing variants (N = 4) and in-frame insertions/deletions (N = 7) were identified to a lesser extent.

After systematic variant reclassification, 83 unique variants in *TSHR*, *DUOX2*, *TPO*, and *TG* genes were classified as P, LP, or VUS_LP, and considered here of clinical relevance (**Table 3**). On the other hand, 30 variants in those genes were identified as not clinically relevant. After reclassification, the *TSHR* remained as the gene with more disease-causing variants (N = 45).

We could identify recurrent variants, appearing in three or more individuals, only in the *DUOX2* and *TSHR* genes (**Supplementary Table S6.5**). The clinically relevant variants NP_000360.2:p.Arg450His, p.Cys41Ser, and p.Pro162Ala in the *TSHR* gene were reported in 21, 14, and 13 cases with MIH, respectively. The variant NP_001350640.1:p.Lys530* in *DUOX2* was reported in 5 individuals.

The relative frequency of each genotype was analyzed, taking into consideration only putative disease-causing variants (**Table 4**). The relative frequency of monoallelic *TSHR* variant carriers was 63%. As for homozygotes and compound heterozygotes, the relative frequency was 8% and 4%, respectively. The frequency of *DUOX2* variant carriers was 6% for heterozygotes, and 7.4% for homozygotes and compound heterozygotes in this dataset. Overall, variants of clinical relevance in the *TSHR* and *DUOX2* genes can explain the MIH phenotype in these 121 cases. We found only a few cases carrying presumed disease-causing variants in the *TG* and *TPO* genes. Monoallelic variants in *TG* or *TPO* were reported in 8 cases. Only three cases were reported to have compound heterozygous variants in *TPO*. Finally, we found three cases of presumed oligogenic inheritance (**Supplementary Table S6.6**).

**Table 4:** Genotype summary after reclassification.

Genotype	*TSHR*	*DUOX2*	*TPO*	*TG*	All
All	111 (77)	22 (15)	8 (5)	4 (3)	145†
Het	92 (63)	9 (6)	4 (3)	4 (3)	109 (75)
Comp_Het	6 (4)	11 (6)	3 (2)	0 (0)	20 (14)
Homo	11 (8)	2 (1.4)	0 (0)	0 (0)	13 (9)
Oligo	2 (1.4) ^1 2^	0 (0)	1 (0.7) ^3^	0 (0)	3 (2)

The numbers represent the count of cases carrying clinically relevant variants (reclassified as P, LP, or VUS_LP) according to each genotype. In parenthesis the proportion (%). *TSHR*, thyroid-stimulating hormone receptor; *TPO*, thyroid peroxidase; *TG*, thyroglobulin; *DUOX2*, dual oxidase 2; Het, Heterozygous Monogenic; Comp_Het, Compound Heterozygous Monogenic; Hom, Homozygous; Oligo, Oligogenic. ^1^ *TSHR*/*TPO* ^2^ *TSHR*/*DUOX2* ^3^ *TPO*/*TG*. Oligogenic cases were counted only once. † Clinically relevant, monoallelic variants were also reported in 3 patients in the following genes: *GLIS3*, *SLC26A4*, and *PAX8* (not shown).

It is worth noting that most included studies were conducted using the candidate gene strategy. Therefore, we then conducted an explorative subgroup analysis to unravel the genomic landscape of MIH derived from NGS studies. **Supplementary Table S6.7** shows the count of cases carrying clinically relevant variants according to each genotype. The overall relative frequency of monoallelic *TSHR* carriers drastically drops to 15%. No cases of alleged oligogenic inheritance were reported using the NGS approach.

Of note, among patients who received levothyroxine treatment, 64% and 36% were mono and biallelic carriers, respectively (**Supplementary Table S6.8**). Interestingly, 98% of the patients who did not receive treatment were heterozygous carriers.

## 4 Discussion

Molecular diagnosis was highly suggested in 4/10 patients, indicating a modest diagnostic performance for MIH with a 13-gene virtual NGS panel.

The proportion of patients with GIS who receive a molecular diagnosis varies widely across studies. Factors contributing to this variability include differences in patient phenotypes, clinical characterization of the patients, and ethnicity, but especially differences in variant classification and interpretation of the genetic test. Although the ACMG/AMP guidelines established a widely adopted five-tier system and a framework of evidence-based criteria for classifying variants in clinical practice (32), a consensus-structured standard that ensures evidence-based classifications is currently missing for most gene-disease or gene-phenotype associations. Our work represents an important, initial step in further facilitating the interpretation of genomic data from MIH. We proposed a variant classification framework and genetic diagnostic criteria for MIH. Furthermore, we provided the research community with a unique and accurately annotated database of MIH variants, mapped to functionally relevant transcripts in GRch37 to facilitate future investigations.

Different studies have reported monoallelic variants in TDH using NGS technology (26). The prevailing understanding is that most cases of TDH are inherited in an autosomal recessive manner; however, hypotheses have been raised about the heritability of CH, especially CH associated with variants in *TSHR* and *DUOX2* genes, which appear to follow autosomal recessive and autosomal dominant patterns (10). Isabelle Oliver-Petit and col. have recently reported heterozygous variants in the *TG*, *TPO*, *DUOX2*, and *TSHR* genes associated with moderate and mild CH (11).

In several reports, *TSHR* variants were regarded as an autosomal dominant trait (38, 39). Almost two decades ago, it was suggested for the first time that a mechanism of negative dominance by direct interaction of the *TSHR* mutant with wild-type receptors as an explanation for the dominant inheritance of partial TSH resistance (38). Different authors have reported that patients with monoallelic *TSHR* variants exhibited a heterogeneous clinical presentation ranging from subclinical to severe CH (11, 40). Patient 9 is illustrative of a chronically slightly elevated TSH value associated with a monoallelic *TSHR* variant.

*TG* or *TPO* deficiency is generally inherited in an autosomal recessive manner, and affected patients have either homozygous or compound heterozygous variants, so thyroid dysfunction is not expected in heterozygous individuals. However, there are some reports of TDH caused by monoallelic variants in *TPO* or *TG* (11, 41). Fugazzola L (2003) reported a case of three siblings with severe CH due to a total iodine organification defect caused by a monoallelic *TPO* variant (42). Nicholas AK (2016) has reported four cases of subclinical or mild CH, classified as unsolved or ambiguous, harboring heterozygous sequence variants in *TG* or *TPO* (10). One study recently reported monogenic heterozygous *TG* variants in three patients. The authors demonstrated that the pathogenic variation co-segregated with the phenotype in one family, suggesting it is possibly causative for CH (14).

We presented here patient 5 with a monoallelic novel variant in the *TG* gene. Excluding this important finding, which has also expanded the *TG* variation spectrum, a single rare variant is not sufficient to explain the pathophysiology of MIH, and the case remains unsolved.

Our systematic review showed that monoallelic clinically significant variants in *TG* or *TPO* were rarely reported (N = 8, **Table 4**). Conversely a ~70% of monoallelic variants of clinical relevance in the *TSHR* and *DUOX2* genes reported to be associated with MIH may explain the disease genetically.

In a model of complete penetrance and expressivity, the pathogenetic variant should co-segregate with the phenotype in the family; however, the diagnosis of MIH is determined mainly by laboratory evaluations, and most patients exhibit few or no signs or symptoms of thyroid dysfunction. The observation that some maternal *DUOX2* mutation-carriers are euthyroid in adulthood supports the transiency of CH in most affected cases (19). Higher TSH screening cut points 20 years ago may have failed to diagnose borderline CH in carrier parents.

In our systematic review of the literature, we collected information about the family history of thyroid disease and segregation analyses when available. Co-segregation with phenotype was verified in 5 compound heterozygous patients. On the other hand, 34 monoallelic variants were also detected in affected parents or siblings, suggesting their pathogenicity in the heterozygous state.

Monoallelic variants have previously been described in association with TDH but are usually assumed to coexist with an additional undetected CNV, intronic, or regulatory variant on the other chromosome. Technical limitations of NGS could explain the absence of a second variant in these patients (25).

Variable expressivity (clinical or phenotypic heterogeneity) in cases harboring similar causative variants suggests that mono- and oligogenic factors, as well as environmental modulators, may play a role in determining disease severity (10). Endocrine disruptors and environmental factors (e.g., iodine intake, acquired thyroid disorders, and drugs affecting thyroid function) could contribute to endocrine dysfunction by influencing gene expression and generating a more profound phenotype in carriers of rare genetic variants (9). Epigenetic factors (11) and autosomal monoallelic expression could not be excluded.

Recent NGS studies propose that digenic or oligogenic effects may play an important role in the pathogenesis of TDH (10, 14, 17). (9, 11, 13, 18) Similarly, oligogenicity is proposed for other endocrine diseases such as Congenital Hypogonadotropic Hypogonadism (43).

In contrast to the monogenic inheritance hypothesis, the true digenic inheritance refers to the phenomenon where the phenotypic expression of a disorder is influenced by disease-causing variants in two different genes. In true digenic inheritance, the simultaneous presence of variants in two genes is necessary to manifest a particular phenotype or disease (44). To account for variable expressivity, some authors have invoked the concept of genetic modifiers, which are considered, in general, to operate as additional molecular defects that determine the final phenotype on the background of the primary pathogenic variant. It has been suggested that multiple partial enzyme deficiencies may lead to clinically relevant biochemical derangements - synergistic heterozygosity - in CH (11). Aycan Z and col. have described for the first time cases with digenic *DUOX1/DUOX2* variants causing complete DUOX isoenzyme deficiency in the context of likely iodine deficiency. These individuals manifest severe CH, suggesting failure to compensate for defective thyroid H2O2 synthesis (45). Yang R and col. have recently reported 10 cases carrying digenic variants in genes involved in TDH, along with a literature review on digenic variants recording 58 cases with CH (28). One hundred and eighteen combinations are linked to CH in the OLIgogenic diseases DAtabase (OLIDA, https://olida.ibsquare.be, last accessed September 2025), a curated database of oligogenic diseases and their gene variants.

In this study, presumed digenic or pseudo-digenic inheritance was detected in three cases from the pilot cohort study. A definite diagnosis was established in patient 6 based on our genetic diagnosis criteria (**Supplementary Table S4**) when the monoallelic variant classified as LP in the *DUOX2* gene is considered (NM_001363711.2:c.2895_2898del). However, assuming a monogenic plus modifier scenario, the P variant in the *TG* gene can be regarded as a putative genetic modifier (NM_003235.5:c.886C>T). The primary disease-causing variant and the modifier together may better explain the clinical picture of the patient than each variant alone. Similarly, the phenotype in patients 2 and 8 is explained by a heterozygous variant in the *TSHR* gene in combination with a modifier in the *TPO* gene. Through systematic review we have identified three cases of likely oligogenic inheritance associated with MIH (**Supplementary Table S6.6**). Interestingly, in two individuals, monoallelic variants in the *DUOX2* or *TSHR* genes can be reasoned as the primary disease-causing variants in a presumed pseudo-digenic scenario. In the third patient, a deleterious variant combination was reported in the *TPO* and *TG* genes, but probably more evidence is needed to establish a true digenic model.

Further research is needed to clarify the role of rare *TG* and *TPO* variants in digenic inheritance or if they may act as disease modifiers of the phenotype. In general, more studies with large pedigrees and clear phenotypic variability investigating oligogenic involvement in CH are required (25, 40).

Digenic variants appeared to be common in CH, but they challenge variant interpretation and make clinical diagnosis difficult. The analysis of oligogenic combinations is currently not subjected to any standards and guidelines and requires different considerations from the framework developed for the interpretation and reporting of variants implicated in Mendelian diseases.

The treatment of CH is not conditional on identifying genetic etiology. However, personalized treatment strategies should consider the individual genetic background, potential compensatory mechanisms, and overall, the clinical context, to optimize care for all patients in the broad spectrum of thyroid hypofunction. A recent study reported that genetic testing influenced treatment decisions for patients with permanent CH (14).

Uncertainty about the benefits of levothyroxine therapy still exists for partial resistance to TSH and compensated euthyroid MIH in children (7, 46). L-T4 treatment seems reasonable in patients with childhood SCH who have TSH levels >10 µU/ml during the first 3 years of life when most thyroxine-dependent brain maturation has occurred (46). Then, a follow-up plan should be tailored to the specific circumstances of the child. Leonardi et al. have stated that when serum TSH is higher than normal during early childhood, a high risk (~50%) of persistent SCH is present in children with genetic and/or morphological abnormalities and should be reassessed periodically (47). Previous studies have shown that untreated children with well-compensated hyperthyrotropinemia due to monoallelic *TSHR* genetic alterations have normal growth and development (48–50). Tenenbaum-Rakover et al. studied the long-term outcome (over 11 years) of loss-of-function variants in the *TSHR* gene and showed that SCH in heterozygous subjects is a stable compensated condition with an appropriately adjusted pituitary TSH set point and does not require replacement therapy (51). However, homozygous and compound heterozygous subjects may necessitate L-T4 therapy because of incompletely compensated SCH (49, 51).

Genetic information allows the individualized decision to follow up without treatment for mildly elevated TSH in childhood. For patients with a definitive or inconclusive molecular diagnosis, such as our case study, periodic biochemical surveillance through puberty, young adulthood, and pregnancy should be implemented to ensure early identification of patients who might benefit from treatment. Patients and mutation-carrier family members should be counseled regarding the possible future risk of overt hypothyroidism, particularly during pregnancy, when iodine status can also be compromised. Furthermore, patients with MIH could evolve into clinical hypothyroidism in case of acquired thyroid disorders, such as the onset of autoimmunity (46). Finally, familial genetic counseling may also help in recognizing the threat of CH in the event of compound heterozygous carriers. There are some reports of cases carrying a heterozygous *TSHR* variant associated with adult-onset compensated TSH resistance having compound heterozygote children with CH (52).

This study has some limitations that should be considered. The main restriction of our study is the limited number of patients. Yet, as our hospital operates as a referral medical facility specializing in the treatment of CH, the results still hold a certain degree of representation. Samples were obtained from parents for segregation analysis; however, co-segregation of the variant with the disease phenotype could not be verified in all cases. Iodine status was not assessed. The requirement for ongoing levothyroxine replacement (case 7) or continuing TSH elevation (cases 1 and 3) suggested persistent MIH in at least three unsolved cases. Two other unsolved cases have not yet undergone a formal trial-off levothyroxine withdrawal (patients 4 and 10). It is also conceivable that despite adequate median coverage, nonuniform coverage of genes could have failed to detect variants (type II error). It was verified that, in general, the coverage of the exons of interest at 20x is at least 99%, and uncovered regions still have a coverage of 10x >90%. No functional analysis was carried out in this study to evaluate the mechanism by which monoallelic variants resulted in functional impairment. Finally, beyond inherent limitations of the systematic review process, the major constraint we encountered was the differing sequencing methodologies, with a relatively low number of cases investigated with the NGS approach.

## 5 Conclusions

Our study provides new insights into the genetic etiology of MIH and expands the phenotypic and variant spectrum of CH. The pilot cohort demonstrated a modest diagnostic yield using a 13-gene NGS panel, while the systematic review offers a comprehensive resource of clinically relevant variants, with *TSHR*, *DUOX2*, *TPO*, and *TG* being the most frequently affected genes. The overall results suggest that the MIH phenotype is influenced not only by monoallelic and biallelic variants but also potentially by digenic variant combinations. Further studies are required to delineate the clinical significance of monoallelic variants in MIH, especially in *TG* and *TPO* genes.

Overall, these findings highlight the importance of comprehensive genetic testing for personalized follow-up and management and emphasize the need for larger multicentric prospective studies to investigate the utility of NGS in guiding treatment decisions and predicting prognosis for MIH patients.

## Data Availability

The raw data from high-throughput sequencing remains confidential due to ethical considerations. All additional raw data supporting the conclusions of this article will be made available by the corresponding author, without undue reservation. The data that support the findings of the systematic review were derived from published papers. The dataset, “Mild Hyperthyrotropinemia Variants Database”, can be found in the Repositorio Institucional CONICET Digital at http://hdl.handle.net/11336/254971.
