# Supplementary material for: Variation spectra in mild isolated hyperthyrotropinemia: pilot cohort and systematic review
Source: Front Endocrinol (Lausanne). 2025 Nov 4;16:1612450. doi: 10.3389/fendo.2025.1612450 (PMC12623176; doi:10.3389/fendo.2025.1612450)
Supplement: Supplementary file 1 [file DataSheet1.docx]

Supplementary Material

**S1. Table “Tailoring the ACMG/AMP 2015 variant classification framework for MIH”**

**S2. Systematic Review - Material and Methods**

**Information source and search strategy**

**Eligibility criteria**

**Selection process and Data extraction**

**Variant reclassification**

**S3. Table “Genetic Diagnosis Criteria”**

**S4. Case Report (Patient 9)**

**S5. Figure “Segregation analysis”**

**S6. Systematic Review – Results**

**6.1 Search and selection process**

**6.2 List of PubMed Identifiers (PMID) of excluded studies**

**6.3 List of PubMed Identifiers (PMID) and DOI of included studies**

**6.4 Flowchart of manuscript selection**

**6.5 Table “Recurrent variants”**

**6.6 Table “Oligogenic combinations”**

**6.7 Table “Number of cases according to genotype in the NGS dataset”**

**6.8 Table “Number of cases according to genotype and levothyroxine treatment”**

**S1.** **Table “Tailoring the ACMG/AMP 2015 variant classification framework for MIH”**

| **“Null variant”** (PVS1) |
| --- |
| Use as defined by ClinGen SVI working group. (1) (2)  Suggestions:   - Use NMDEscPredictor (https://nmdprediction.shinyapps.io/nmdescpredictor/) to estimate if a frameshift variant is predicted to escape nonsense-mediated decay (NMD). - Verify that the exon is present in a biologically relevant transcript, the MANE (Matched Annotation from the NCBI and EMBL-EBI) Select transcript for this gene, in gnomAD. - Verify in gnomAD that Loss-of-function (LoF or null) variants (including nonsense variants, splice site variants, frameshift variants, and deletions) in the exon are not frequent in the general population (Other databases: ClinVar, Simple ClinVar, DECIPHER). - AutoPVS1, an automatic classification tool, can be used for PVS1 interpretation (https://autopvs1.bgi.com/).(3) |
| **“Other Nucleotide” or “Different Missense”** (PS1 or PM5) |
| No specific recommendations for PS1 (variant create same amino acid change as a known pathogenic variant), and PM5 (novel missense variant at the same position as known pathogenic variant). (4)  Applicable if SpliceAI does not predict any change in splicing of the new base pair change. |
| ***“De Novo* with/without parental testing”** (PS2 and PM6) |
| Use as recommended by the ClinGen SVI WG [[PS2/PM6: Recommendation for de novo PS2 and PM6 ACMG/AMP criteria (Version 1.1)](https://clinicalgenome.org/docs/ps2-pm6-recommendation-for-de-novo-ps2-and-pm6-acmg-amp-criteria-version-1.0/)]. |
| **“Functional Studies”** (PS3/BS3) |
| Use PS3/BS3 according to the recommendations provided by the ClinGen SVI WG. (5)  Since validation controls and replicates are rarely documented for functional assays in CH, the functional data will initially be considered supporting level evidence. |
| **“Prevalence in affected individuals”** (PS4) - Case-level data |
| Resistance to thyroid-stimulating hormone (OMIM # 275200) and thyroid dyshormonogenesis-6 (OMIM # 607200) are considered autosomal dominant traits.  Use PS4 (variant prevalence being significantly increased in affected individuals compared to controls) as recommended by the ClinGen Cardiomyopathy Variant Curation Expert Panel for the MYH7 gene. A case-counting approach is applied and weighted according to the number of probands with that variant (Supporting: ≥2; Moderate: ≥6; Strong: ≥15).  Importantly, only unrelated probands with a phenotype fitting the disease are counted, and the variant should meet PM2_supporting. |
| **“Functional Domain Hot Spot”** (PM1) |
| Applicable for missense variants within a functionally critical region of a protein, and therefore a mutational hotspot with very little/no benign variation (PM1_moderate) or with some benign variation (PM1_supporting).  Suggestions:   - Structural Studies: Use 3-dimensional protein modelling to precise positioning of a variant within the functional region of a protein and to get mechanistic insights about how a variant alters domain function. - Protein Domains: For checking critical functional domains use InterPro (<https://www.ebi.ac.uk/interpro/>), UniProt (<https://www.uniprot.org/>) and Decipher (<https://www.deciphergenomics.org/>). - Hotspots and Benign Variation: For evidence of local enrichment of pathogenic missense variation, use ClinVar (<https://www.ncbi.nlm.nih.gov/clinvar/>), gnomAD (<https://gnomad.broadinstitute.org/>), and the University of California Santa Cruz (UCSC) Genome Browser (<https://genome.ucsc.edu/>). - Multiple sequence alignments: To predict if a residue lies in an invariant position, use Pfam data available through InterPro and HMMER web server (<https://www.ebi.ac.uk/Tools/hmmer/>). |
| **“Allele Frequency”** (PM2) |
| For variants having a minor allele frequency (MAF) <0.1% in the Genome Aggregation Database (gnomAD) (6), the criterion PM2_supporting is applied, according to the last ClinGen Sequence Variant Interpretation (SVI) Recommendation for PM2 [[PM2: Recommendation for Absence/Rarity Criterion PM2 (Version 1.0)](https://clinicalgenome.org/docs/pm2-recommendation-for-absence-rarity/)].  Of note, monoallelic variants in genes such as *DUOX2, TG, TPO* and *TSHR* may occur in the general population, and Mild Isolated Hyperthyrotropinemia (MIH) may evade diagnosis on neonatal Congenital Hypothyroidism (CH) screening in most countries, such that “apparently healthy” mutation carriers satisfy criteria for inclusion in the population genetic databases such as gnomAD. (7) (8)  Average coverage of sequenced regions should be at least 20x. |
| **“In trans with a pathogenic variant”** (PM3) |
| Thyroid dyshormonogenesis-2A (OMIM **#** 274500) and thyroid dyshormonogenesis-3 (OMIM **#** 274700), are considered recessive traits.  Use PM3 according to the ClinVar SVI point system [[PM3: Recommendation for in trans Criterion PM3 (Version 1.0)](https://clinicalgenome.org/docs/pm3-recommendation-for-in-trans-criterion-pm3-version-1.0/)].  The proband must be affected. The variant should meet PM2_supporting. |
| ***“In-frame* deletions/insertions”** (PM4/BP3) |
| Use as defined in “ACGS Best Practice Guidelines for Variant Classification in Rare Disease 2024” (https://www.acgs.uk.com/quality/best-practice-guidelines/).  If the region does not show evolutionary conservation, PM4 should not be used. |
| **“Segregation Data”** (PP1/BS4) |
| Apply PP1 criterion (cosegregation in affected family members) to the prioritized variants in the index case, based on co-segregation analysis following recommendations from Biesecker et al. 2024. (9)  Of note, the diagnosis of MIH is determined mainly by laboratory evaluations, and most family members may exhibit few or no signs or symptoms of thyroid dysfunction. The blood TSH levels of first-grade relatives should be tested. |
| **“Missense Variants”** (PP2) |
| Only applicable for genes with a missense Z score (gnomAD) ≥ 3.09.  MIH-associated genes, *TSHR*, *DUOX2*, *TG,* and *TPO* with a Z-score of less than 3.09, are considered tolerant. |
| **“Computational evidence”** (PP3/BP4) |
| Use PP3 for missense variants predicted deleterious by REVEL (rare exome variant ensemble learner), an ensemble method for predicting the pathogenicity of missense variants. (10)  Use threshold ranges according to Pejaver V (2022). (11)  Also applicable for missense, intronic and noncoding variants with predicted impact on splicing.  SpliceAI is used for splice variants. (12) Noncanonical splice sites (NCSSs) variants are selected if the delta score was >0.2.  Not applicable for canonical splice variants.  For *in-frame* indels, apply the PP3 criterion if at least two of three tools, PROVEAN (Protein Variation Effect Analyzer, <https://www.jcvi.org/research/provean>), MutationTaster (13), and MutPred-InDel (14), indicate a deleterious prediction. The reliability of these tools has been demonstrated by Cannon et al. (2023) et al, who have assessed the performance of various pathogenicity prediction algorithms for *in-frame* indels. (15) |
| **“Highly Specific Phenotype”** (PP4) - Case-level data |
| Use PP4_supporting for sequence alterations in the *TSHR* gene given the extent of evidence on phenotype specificity. |
| **“Reputable source”** (PP5/BP6) |
| PP5/BP6 are not used according to recent recommendations (Biesecker 2018). (16) |
| **“Benign Stand-Alone”** (BA1), “**Allele frequency is greater than expected”** (BS1), **“In healthy adults”** (BS2) |
| No specifications. |
| “**Missense variant in a gene with a LoF mechanism”** (BP1), “**Explained by another pathogenic variant”** (BP2), “**Alternate Locus”** (BP5) |
| No specifications. |
| **“Synonymous Variant”** (BP7) |
| No specific recommendations for PB7. (4)  Synonymous variants with a SpliceAI prediction score below 0.1 were assigned BP7. |

**S2. Systematic Review - Material and Methods**

**Information source and search strategy**

The literature search was done on studies up to 8 Jan 2025 on PubMed database from the National Library of Medicine using the following keywords and terms: (congenital OR neonatal OR newborn OR child OR children OR infant OR toddler) AND ("subclinical hypothyroidism" OR "compensated hypothyroidism" OR hyperthyrotropinemia OR hyperthyreotropinemia OR hyperthyrotrophinaemia OR hyperthyrotropinaemia OR "resistance to thyrotropin" OR "resistance to TSH" OR "TSH resistance" OR "thyrotropin resistance" OR (mild AND "congenital hypothyroidism")) AND (sequencing OR mutation OR variant).

**Eligibility criteria**

Eligible studies were limited to those published in English. Articles were screened using the following inclusion criteria: 1) include cases identified by authors as MIH or subclinical hypothyroidism (SCH) and/or the biochemical phenotype can be inferred, preferably with data on TSH and T4 and/or FT4 levels (moderately elevated serum TSH levels with a normal T4 and/or FT4 level for age), and 2) the MIH has been diagnosed during the neonatal period (3 months) or childhood (18 years). The TSH cut-off value for inclusion was set arbitrarily at 22 µU/ml considering a variability of 10%. Subjects with goiter or mild hypoplasia were included if they met the inclusion criteria.

Cases presenting 1) T4 and/or FT4 in the normal range with a higher level of TSH (>22 µU/ml) referred to as mild CH, compensated hypothyroidism, or mild-severe TSH resistance, 2) positive antithyroid antibodies, 3) TSH levels >22 µU/ml at any time, e.g. after L-T4 discontinuation, and/or 4) evidence of severe thyroid dysgenesis (i.e., thyroid aplasia, ectopia, or hypoplasia), were excluded. Excluded were also studies reporting synonymous SNVs, common sequence variants (“genetic polymorphisms”), or variants classified as benign or likely benign by authors. Finally, we excluded review articles and studies reporting only *in vitro* experiments.

**Selection process**

VR and MT reviewed titles and abstracts of all records to identify those that would be included in the full-text review. When there was disagreement, a consensus discussion also included MM. Full text and supplementary data were assessed to select studies.

**Data extraction**

Data collection was performed independently by VR and MLT, with any disagreements regarding the inclusion of citations being referred to MM for mediation. For family studies with different cases meeting eligibility criteria, data regarding one family member who meets the inclusion criteria, usually the index case, was included. No attempts were made to contact the authors of the original articles for information; however, previously published data was occasionally extracted from citations when available.

Information was collected using a standardized data collection form from the main manuscript and supplementary material.

Information was extracted from the published studies, along with the article's ID according to PubMed (PMID) and patient ID. Demographic features (age and gender), biochemical parameters, and imaging findings were recorded. Information of presumably pathogenic or causative variants was extracted from the published studies. We collected the targeted genes and the diagnostic strategy (Sanger sequencing, NGS, etc.), gene name, gene variant (variant coding DNA nucleotide change and/or amino acid change, as given by the authors), zygosity (homozygous, heterozygous).

Data not available was registered as such (NAV).

The risk of bias assessment was not performed because 2 of the 3 categories of the NEWCASTLE-OTTAWA QUALITY ASSESSMENT SCALE for COHORT STUDIES (Comparability and Outcome) are not applicable in this study.

**Variant reclassification**

The list of variants was assembled in an Excel file and mapped onto the human reference genome GRCh37/hg19. Variants with insufficient data for remapping were removed due to uncertainty. The pathogenic potential of the variants was systematically reevaluated with Franklin by Genoox (<https://franklin.genoox.com/>: Last Accessed January 2025). Franklin generates an automated, suggested classification for each input variant. Franklin employs an internal seven-tier classification system where VUSs are divided into VUS-Leaning-Pathogenic (VUS-LP), VUS-Leaning-Benign (VUS-LB), and VUS-VUS.

**Synthesis method**

To provide an overview of the genes and variants associated with MIH, we described the proportion of affected patients for each gene according to genotype after having reclassified the sequence variants. We also performed subgroup analysis of data derived from NGS studies. We separately analyzed data from studies which implemented targeted NGS or WES (“NGS dataset”). The “broad dataset” includes both studies using the candidate gene approach (hypothesis-based) and NGS.

**Registration and protocol**

The review protocol can be accessed on request. The review was not registered (PROSPERO automatically rejects records if risk of bias was not performed).

**S3. Case report**

Patient 9, the third child of healthy non-consanguineous Caucasian Argentinian parents, was a female born at term after an uneventful pregnancy and delivery. Her family history was negative for thyroid dysfunction. Neonatal screening for congenital hypothyroidism revealed a TSH level of 17 µU/ml on the 2nd day of life and 15 µU/ml on the 10th day. She was asymptomatic with a normal physical examination. A serum sample obtained at 1 month of age showed elevated TSH (TSH= 19.97 µU/ml) and Tg with normal thyroid hormones; anti-TPO antibody and anti-Tg antibody were negative. A thyroid scan using Tc99 showed the presence of an eutopic enlarged gland. She was treated with L-T4 and serum TSH was subsequently maintained in the normal range with a reduced dose. At 3 years and 9 months of age, treatment was withdrawn for re-evaluation, after which MIH was documented (TSH= 13.87 µU/ml) and L-T4 resumed. The thyroid US was normal at this time. There were no recognizable abnormalities in growth, intelligence, and pubertal development, with menarches occurring at 12 years of age. She performed well academically. At 13 years and 6 months, L-T4 treatment was discontinued. Afterwards, serum TSH levels were monitored periodically, with values fluctuating between 4.96 and 9.2 µU/ml. The patient remained asymptomatic, although she reported oligomenorrhea during periods of psychological stress.

**S4. Table “Genetic diagnosis criteria”**

| **Definitive molecular diagnosis*** |
| --- |
| • 2 P/LP variants (in trans) in *TSHR*, *DUOX2*, *TG* or *TPO*  • 1 P/LP variant in *TSHR* or *DUOX2* |
| **Inconclusive-Possibly solved** |
| • 1 or 2 (in trans) VUS_LP in *TSHR* or *DUOX2*  • 1 P/LP variant in *TG* or *TPO +* 1 VUS_LP in *TG* or *TPO* (in trans, monogenic) |
| **Inconclusive** |
| • 1 P/LP variant in *TG* or *TPO +* 1 VUS_LP in *TG* or *TPO* (in trans, oligogenic)  • 1 P/LP/VUS_LP variant in *TG* or *TPO*  • 2 VUS_LP variants in *TG* or *TPO* (in trans, mono or oligogenic)  • 1 or 2 P/LP/VUS_LP in other genes associated with TDH (in trans, mono or oligogenic) |
| * According to ACMG/AMP guidelines, only variants classified as P or LP are considered definitive for a genetic diagnosis. P: pathogenic. LP: likely pathogenic. VUS_LP: variant of uncertain significance leaning to pathogenic. VUS: variant of uncertain significance. VUS_LB: variant of uncertain significance leaning to benign. LB: likely benign. B: benign. |

**S5. Figure “Segregation analysis”**

Sanger sequencing and segregation analysis were performed to validate the identified variants in affected individuals and their parents when available.


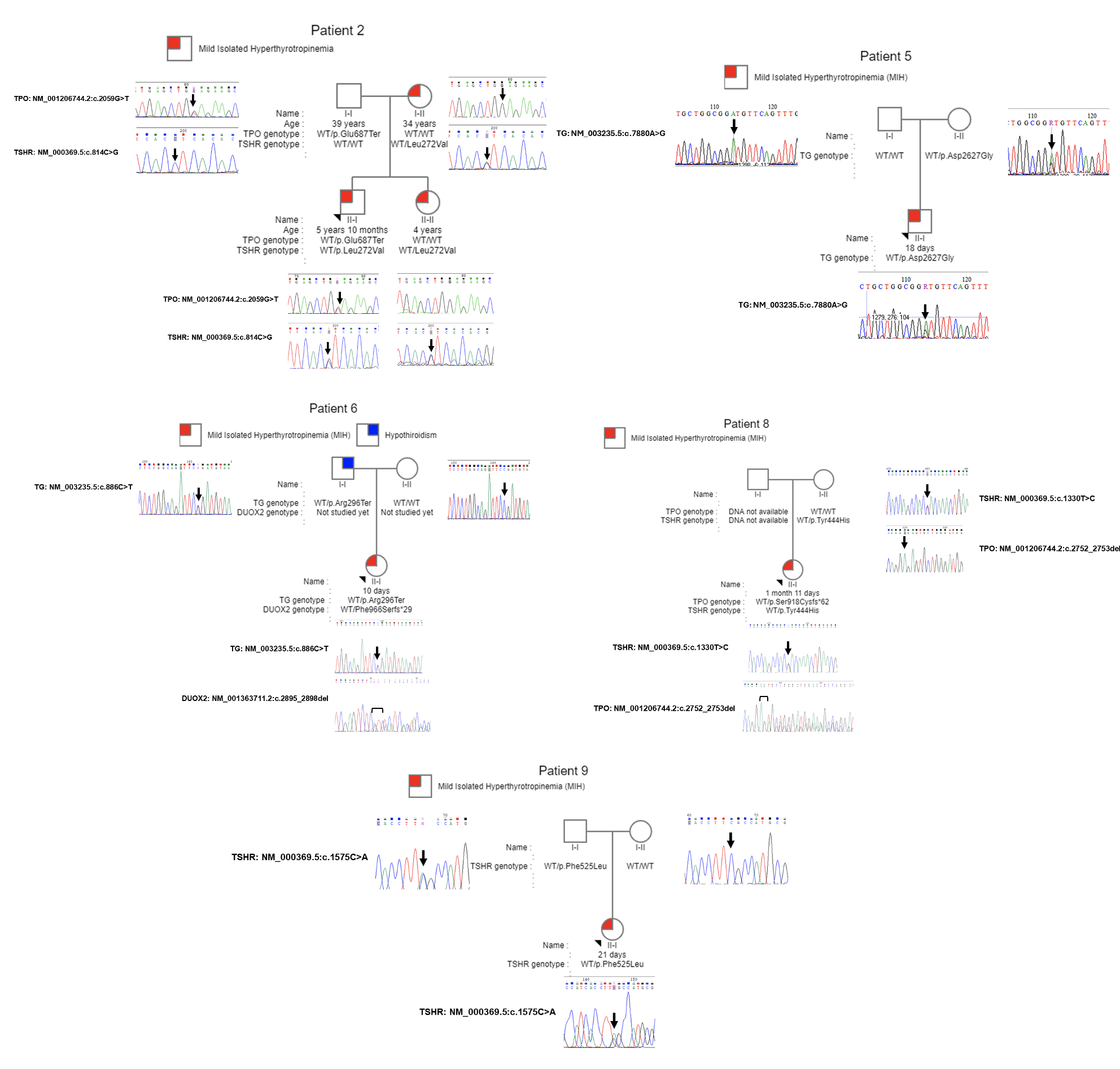


**S6. Systematic Review – Results**

**S6.1 Search and selection process**

The search through PubMed yielded 233 citations. After screening article titles and abstracts, 69 articles were identified for full-text retrieval and review. Sixty-six additional records were identified from citation searching. Four full texts could not be retrieved. One hundred thirty-four manuscripts and supplementary data were carefully examined to ensure they reported variants with appropriate sample information and/or genome mapping details for each eligible case. A total of 90 articles were excluded (S6.2). Especially, we excluded duplicated publications of the same patients, based on sample ID and/or variant characteristics, and articles with poor reporting of patient phenotype or genotype (i.e., lacking the information necessary to remap and annotate the gene variant). Finally, 44 articles were included. The PubMed Identifiers (PMID) of the included studies are available at S6.3. The reader may refer to the manuscripts for specific information about study design. Clinical characteristics of the included cases are available at the “Mild Isolated Hyperthyrotropinemia Variants Database”.

The search strategy is illustrated in Figure S6.4.

**S6.2 List of PubMed Identifiers (PMID) of excluded studies**

1752952

7528344

8954020

9100579

9588493

9924196

11061528

11095460

11415848

11549705

12110737

14725684

15531543

15671778

15807875

15870119

16117192

16135555

17456567

17532758

18042646

18445672

18631008

19158199

19169484

19169491

19189706

19438905

19837936

19916865

20410234

20447071

20972728

21186955

21367925

21677043

21714469

21958696

22784463

23164529

23292166

23426615

23455760

23563316

23933148

23949896

24423310

24735383

25146893

25263060

25633667

25675383

25928756

26316439

26709262

26777470

26831560

27108200

27373559

27525530

28215547

28444304

29275168

29650690

30083029

30375286

31030636

31044655

31356790

31430255

31867598

32088313

32425884

32930933

33298898

33321114

33653783

34456971

34632506

34780050

35507000

36125728

36547798

37164149

37346832

38923290

39003160

39040671

39189676

39337518

**S6.3 List of PubMed Identifiers (PMID) and DOI of included studies**

| PMID | DOI |
| --- | --- |
| 12050212 | 10.1210/jcem.87.6.8536 |
| 12107226 | 10.1210/jcem.87.7.8662 |
| 12864797 | 10.1046/j.1365-2265.2003.01823.x |
| 15693879 | 10.1111/j.1442-200x.2005.02020.x |
| 16060907 | 10.1111/j.1365-2265.2005.02314.x |
| 16134168 | 10.1002/humu.9372 |
| 16320156 | 10.1055/s-2005-865914 |
| 16756469 | 10.1089/thy.2006.16.471 |
| 17468186 | 10.1530/EJE-07-0037 |
| 17524032 | 10.1111/j.1365-2265.2007.02877.x |
| 17526952 | 10.1007/s12020-006-0018-z |
| 17697008 | 10.1111/j.1365-2265.2007.02950.x |
| 18426362 | 10.1089/thy.2007.0258 |
| 19240155 | 10.1210/jc.2008-1938 |
| 19417038 | 10.1210/jc.2009-0375 |
| 19506388 | 10.1159/000223415 |
| 19509106 | 10.1210/jc.2009-0150 |
| 19820021 | 10.1210/jc.2009-0618 |
| 21490078 | 10.1210/jc.2011-0127 |
| 21565790 | 10.1210/jc.2010-2467 |
| 21900383 | 10.1210/jc.2011-1573 |
| 22049173 | 10.1210/jc.2011-1938 |
| 23332130 | 10.1186/1824-7288-39-5 |
| 23412867 | 10.1515/jpem-2012-0308 |
| 23926367 | 10.1297/cpe.18.95 |
| 25248169 | 10.1159/000362235 |
| 25557138 | 10.1089/thy.2014.0311 |
| 26742565 | 10.1530/EJE-15-0959 |
| 27166716 | 10.1515/jpem-2015-0400 |
| 27637299 | 10.1016/j.cca.2016.09.007 |
| 27821020 | 10.1089/thy.2016.0469 |
| 28561265 | 10.1111/cen.13387 |
| 28867693 | 10.1507/endocrj.EJ17-0194 |
| 29092890 | 10.1530/EJE-16-1049 |
| 32078117 | 10.1007/s12020-020-02224-5 |
| 32459320 | 10.1210/clinem/dgaa308 |
| 32469330 | 10.1515/jpem-2019-0433 |
| 32765423 | 10.3389/fendo.2020.00413 |
| 34234053 | 10.1507/endocrj.EJ21-0353 |
| 34248839 | 10.3389/fendo.2021.657913 |
| 36913313 | 10.1530/ETJ-22-0212 |
| 38433572 | 10.3343/alm.2023.0337 |
| 39029043 | 10.1097/MD.0000000000038976 |
| 39728398 | 10.3390/ijns10040078 |

**S6.4 Flowchart of manuscript selection**

**S6.5 Table “Recurrent variants”**

| Gene | Variant | N cases | N papers | Links |
| --- | --- | --- | --- | --- |
| TSHR | NP_000360.2:p.Arg450His | 21 | 13 | [22550](http://www.ncbi.nlm.nih.gov/clinvar/variation/225505), [189261858](http://www.ncbi.nlm.nih.gov/snp/189261858), [CA7294442](https://reg.clinicalgenome.org/redmine/projects/registry/genboree_registry/by_caid?caid=CA7294442) |
|  | p.Cys41Ser | 14 | 5 | [6442](http://www.ncbi.nlm.nih.gov/clinvar/variation/6442), [121908869](http://www.ncbi.nlm.nih.gov/snp/121908869), [CA118227](https://reg.clinicalgenome.org/redmine/projects/registry/genboree_registry/by_caid?caid=CA118227) |
|  | p.Pro162Ala | 13 | 6 | [6435](http://www.ncbi.nlm.nih.gov/clinvar/variation/6435), [rs121908863](http://www.ncbi.nlm.nih.gov/snp/rs121908863), [CA118206](https://reg.clinicalgenome.org/redmine/projects/registry/genboree_registry/by_caid?caid=CA118206) |
|  | p.Pro68Ser | 7 | 5 | [437071](http://www.ncbi.nlm.nih.gov/clinvar/variation/437071), [rs142063461](http://www.ncbi.nlm.nih.gov/snp/rs142063461), [CA7294029](https://reg.clinicalgenome.org/redmine/projects/registry/genboree_registry/by_caid?caid=CA7294029) |
|  | p.Arg109Gln | 6 | 5 | [6438](http://www.ncbi.nlm.nih.gov/clinvar/variation/6438), [rs121908865](http://www.ncbi.nlm.nih.gov/snp/rs121908865), [CA118215](https://reg.clinicalgenome.org/redmine/projects/registry/genboree_registry/by_caid?caid=CA118215) |
|  | p.Asp403Asn | 3 | 3 | [2572312](http://www.ncbi.nlm.nih.gov/clinvar/variation/2572312), [rs565082329](http://www.ncbi.nlm.nih.gov/snp/rs565082329), [CA7294409](https://reg.clinicalgenome.org/redmine/projects/registry/genboree_registry/by_caid?caid=CA7294409) |
|  | p.Cys390Phe | 3 | 2 | [2136258](http://www.ncbi.nlm.nih.gov/clinvar/variation/2136258), [rs371139156](http://www.ncbi.nlm.nih.gov/snp/rs371139156), [CA7294399](https://reg.clinicalgenome.org/redmine/projects/registry/genboree_registry/by_caid?caid=CA7294399) |
|  | p.Gln8Hisfs*55 | 3 | 3 | [1204435](http://www.ncbi.nlm.nih.gov/clinvar/variation/1204435), [rs773584994](http://www.ncbi.nlm.nih.gov/snp/rs773584994), [CA7294555](https://reg.clinicalgenome.org/redmine/projects/registry/genboree_registry/by_caid?caid=CA7294555) |
|  | p.Met527Thr | 3 | 3 | [rs1294870139](http://www.ncbi.nlm.nih.gov/snp/rs1294870139) |
|  | p.Thr655Cysfs*2 | 3 | 3 | [1204435](https://www.ncbi.nlm.nih.gov/clinvar/variation/1204435/), [rs761918916](http://www.ncbi.nlm.nih.gov/snp/rs761918916), [CA7294555](https://reg.clinicalgenome.org/redmine/projects/registry/genboree_registry/by_caid?caid=CA7294555) |
|  | p.Trp488Arg | 3 | 3 | [1050267](https://www.ncbi.nlm.nih.gov/clinvar/variation/1050267/?oq=((1038291%5bAlleleID%5d))&m=NM_000369.5(TSHR):c.1462T%3EC%20(p.Trp488Arg)) |
| DUOX2 | NP_001350640.1:p.Lys530* | 5 | 2 | [287079](http://www.ncbi.nlm.nih.gov/clinvar/variation/287079), [180671269](http://www.ncbi.nlm.nih.gov/snp/180671269), [CA7538552](https://reg.clinicalgenome.org/redmine/projects/registry/genboree_registry/by_caid?caid=CA7538552) |
|  | p.Arg1110Gln | 3 | 2 | [420157](http://www.ncbi.nlm.nih.gov/clinvar/variation/420157), [368488511](http://www.ncbi.nlm.nih.gov/snp/368488511), [CA7537915](https://reg.clinicalgenome.org/redmine/projects/registry/genboree_registry/by_caid?caid=CA7537915) |
|  | p.Leu1160del | 3 | 3 | [2725056](http://www.ncbi.nlm.nih.gov/clinvar/variation/2725056), [rs758318135](http://www.ncbi.nlm.nih.gov/snp/rs758318135), [CA7537861](https://reg.clinicalgenome.org/redmine/projects/registry/genboree_registry/by_caid?caid=CA7537861) |

Variants appearing in three or more individuals.

**S6.6 Table “Oligogenic combinations”**

| Gene | Variant* | ACMG/AMP | PubMedID |
| --- | --- | --- | --- |
| *TSHR* (F) | NM_000369.5:c.790C>T | VUS_LP | 21490078 |
| *TSHR* (F) | NM_000369.5:c.269_270inv (AG>CT) | LP |  |
| *TPO* (F) | NM_001206744.2:c.1477G>A | LP |  |
| *DUOX2* (F) | NM_001363711.2:c.979G>T | P | 29092890 |
| *TSHR* (M) | NM_000369.5: c.1349G>A | P |  |
| *TPO* (F) | NM_001206744.2: c.2395G>C | VUS_LP | 32765423 |
| *TG* (M) | NM_003235.5:c.5372del | LP |  |

* All variants were reported in heterozygosity. ACMG/AMP: American College of Medical Genetics and Genomics - Association for Molecular Pathology. *TSHR* (NG_009206.1): thyroid-stimulating hormone receptor. *TPO* (NG_011581.2): thyroid peroxidase. *TG* (NG_015832.2): thyroglobulin. *DUOX2* (NG_009447.1): dual oxidase 2. P: Pathogenic. LP: Likely Pathogenic. VUS_LP: Variant of Uncertain Significance leaning to Pathogenic. (M) maternally inherited. (F) paternally inherited.

**S6.7 Table “Number of cases according to genotype in the NGS dataset”**

| Genotype | *TSHR* | *DUOX2* | *TPO* | *TG* | All |
| --- | --- | --- | --- | --- | --- |
| All | 15 (44) | 14 (41) | 3 (9) | 2 (6) | **34**† |
| Het | 5 (15) | 4 (12) | 1 (3) | 2 (6) | 12 (35) |
| Comp_Het | 3 (9) | 8 (23) | 2 (6) | 0 (0) | 13 (38) |
| Homo | 7 (21) | 2 (6) | 0 (0) | 0 (0) | 9 (27) |
| Oligo | 0 (0) | 0 (0) | 0 (0) | 0 (0) | 0 (0) |

The numbers represent the count of cases carrying clinically relevant variants (reclassified as P, LP, or VUS_LP) according to each genotype. In parenthesis, the proportion (%). *THSR*: thyroid-stimulating hormone receptor. *TPO*: thyroid peroxidase. *TG*: thyroglobulin. *DUOX2*: dual oxidase 2. Het: Heterozygous Monogenic. Comp_Het: Compound Heterozygous Monogenic. Hom: Homozygous. Oligo: Oligogenic. NGS: Next Generation Sequencing. † Clinically relevant, monoallelic variants were also reported in 3 patients in the following genes: *GLIS3*, *SLC26A4*, and *PAX8* (not shown).

**S6.8 Table “Number of cases according to genotype and levothyroxine treatment”**

|  | Levothyroxine | | |
| --- | --- | --- | --- |
| Genotype | Yes | No | NAV |
| Total N (%) | 94 (100) | 28 (100) |  |
| Heterozygous Monogenic | 60 (64) | 27 (96) | 21 |
| Compound Heterozygous Monogenic | 19 (20) | 0 (0) | 1 |
| Homozygous | 13 (14) | 0 (0) | 0 |
| Oligogenic | 2 (2) | 1 (4) | 0 |

NAV: data not available.**Supplementary Material References**

1. Riggs ER, Andersen EF, Cherry AM, Kantarci S, Kearney H, Patel A, Raca G, Ritter DI, South ST, Thorland EC, et al. Technical standards for the interpretation and reporting of constitutional copy-number variants: a joint consensus recommendation of the American College of Medical Genetics and Genomics (ACMG) and the Clinical Genome Resource (ClinGen). *Genetics in Medicine* (2020) 22:245–257. doi: 10.1038/s41436-019-0686-8

2. Abou Tayoun AN, Pesaran T, DiStefano MT, Oza A, Rehm HL, Biesecker LG, Harrison SM. Recommendations for interpreting the loss of function PVS1 ACMG/AMP variant criterion. *Hum Mutat* (2018) 39:1517–1524. doi: 10.1002/humu.23626

3. Xiang J, Peng J, Baxter S, Peng Z. AutoPVS1: An automatic classification tool for PVS1 interpretation of null variants. *Hum Mutat* (2020) 41:1488–1498. doi: 10.1002/humu.24051

4. Richards S, Aziz N, Bale S, Bick D, Das S, Gastier-Foster J, Grody WW, Hegde M, Lyon E, Spector E, et al. Standards and guidelines for the interpretation of sequence variants: a joint consensus recommendation of the American College of Medical Genetics and Genomics and the Association for Molecular Pathology. *Genetics in Medicine* (2015) 17:405–424. doi: 10.1038/gim.2015.30

5. Brnich SE, Abou Tayoun AN, Couch FJ, Cutting GR, Greenblatt MS, Heinen CD, Kanavy DM, Luo X, McNulty SM, Starita LM, et al. Recommendations for application of the functional evidence PS3/BS3 criterion using the ACMG/AMP sequence variant interpretation framework. *Genome Med* (2020) 12:3. doi: 10.1186/s13073-019-0690-2

6. Karczewski KJ, Francioli LC, Tiao G, Cummings BB, Alföldi J, Wang Q, Collins RL, Laricchia KM, Ganna A, Birnbaum DP, et al. The mutational constraint spectrum quantified from variation in 141,456 humans. *Nature* (2020) 581:434–443. doi: 10.1038/s41586-020-2308-7

7. Peters C, Nicholas AK, Schoenmakers E, Lyons G, Langham S, Serra EG, Sebire NJ, Muzza M, Fugazzola L, Schoenmakers N. *DUOX2* / *DUOXA2* Mutations Frequently Cause Congenital Hypothyroidism that Evades Detection on Newborn Screening in the United Kingdom. *Thyroid* (2019) 29:790–801. doi: 10.1089/thy.2018.0587

8. Park K-S. Analysis of Worldwide Carrier Frequency and Predicted Genetic Prevalence of Autosomal Recessive Congenital Hypothyroidism Based on a General Population Database. *Genes (Basel)* (2021) 12:863. doi: 10.3390/genes12060863

9. Biesecker LG, Byrne AB, Harrison SM, Pesaran T, Schäffer AA, Shirts BH, Tavtigian S V., Rehm HL. ClinGen guidance for use of the PP1/BS4 co-segregation and PP4 phenotype specificity criteria for sequence variant pathogenicity classification. *The American Journal of Human Genetics* (2024) 111:24–38. doi: 10.1016/j.ajhg.2023.11.009

10. Ioannidis NM, Rothstein JH, Pejaver V, Middha S, McDonnell SK, Baheti S, Musolf A, Li Q, Holzinger E, Karyadi D, et al. REVEL: An Ensemble Method for Predicting the Pathogenicity of Rare Missense Variants. *The American Journal of Human Genetics* (2016) 99:877–885. doi: 10.1016/j.ajhg.2016.08.016

11. Pejaver V, Byrne AB, Feng B-J, Pagel KA, Mooney SD, Karchin R, O’Donnell-Luria A, Harrison SM, Tavtigian S V., Greenblatt MS, et al. Calibration of computational tools for missense variant pathogenicity classification and ClinGen recommendations for PP3/BP4 criteria. *The American Journal of Human Genetics* (2022) 109:2163–2177. doi: 10.1016/j.ajhg.2022.10.013

12. Jaganathan K, Kyriazopoulou Panagiotopoulou S, McRae JF, Darbandi SF, Knowles D, Li YI, Kosmicki JA, Arbelaez J, Cui W, Schwartz GB, et al. Predicting Splicing from Primary Sequence with Deep Learning. *Cell* (2019) 176:535-548.e24. doi: 10.1016/j.cell.2018.12.015

13. Schwarz JM, Cooper DN, Schuelke M, Seelow D. MutationTaster2: mutation prediction for the deep-sequencing age. *Nat Methods* (2014) 11:361–362. doi: 10.1038/nmeth.2890

14. Pagel KA, Antaki D, Lian A, Mort M, Cooper DN, Sebat J, Iakoucheva LM, Mooney SD, Radivojac P. Pathogenicity and functional impact of non-frameshifting insertion/deletion variation in the human genome. *PLoS Comput Biol* (2019) 15:e1007112. doi: 10.1371/journal.pcbi.1007112

15. Cannon S, Williams M, Gunning AC, Wright CF. Evaluation of in silico pathogenicity prediction tools for the classification of small in-frame indels. *BMC Med Genomics* (2023) 16:36. doi: 10.1186/s12920-023-01454-6

16. Biesecker LG, Harrison SM. The ACMG/AMP reputable source criteria for the interpretation of sequence variants. *Genetics in Medicine* (2018) 20:1687–1688. doi: 10.1038/gim.2018.42
